# NCX2 Regulates Intracellular Calcium Homeostasis and Translocation of HIF-1α into the Nucleus to Inhibit Glioma Invasion

**DOI:** 10.1007/s10528-022-10274-9

**Published:** 2022-11-05

**Authors:** Hongyuan Liu, Ju Yu, Liling Yang, Pengcheng He, Zongping Li

**Affiliations:** 1 Department of Neurosurgery, Mianyang Central Hospital, School of Medicine, University of Electronic Science and Technology of China, Mianyang, People’s Republic of China; 2 Department of Neurosurgery, the Second Affiliated Hospital of Soochow University, Suzhou, People’s Republic of China; 3 Department of Nephrology, Mianyang Central Hospital, School of Medicine, University of Electronic Science and Technology of China, Mianyang, People’s Republic of China

**Keywords:** NCX2, Hypoxic, HIF-1α, Glioma, Invasion

## Abstract

Glioma is the most common tumor of the central nervous system, and its poor prognosis can be linked to hypoxia and gene inactivation. Na^+^/Ca^2+^ exchanger 2 (NCX2) is expressed only in the normal brain and not in other tissues or glioma. We constructed a hypoxic microenvironment to more accurately understand the effect of NCX2 in glioma. Our previous experiments confirmed that NCX2 inhibited the growth of U87 cells in nude mice, indicating that NCX2 is a potential tumor suppressor gene. Malignant tumor cells are often exposed to an anoxic environment. To more accurately understand the effect of NCX2 in glioma, we constructed a hypoxic microenvironment. To detect the localization of NCX2 in transfected U87 cells, immunofluorescence was used. We tested the function of NCX2 in glioma, i.e., how it contributes to the cytosolic Ca^2+^ homeostasis by X-Rhod-1. We tested the cell proliferation of NCX2 in glioma in hypoxic using Cell counting kit-8 (CCK8). Cell migration and invasion were evaluated in 24-well transwell matrigel-coated or non-matrigel-coated in hypoxia. NCX2 promoted the proliferation of U87 cells in the hypoxic microenvironment. It inhibited the invasion and migration abilities of U87 cells. We demonstrated that NCX2 was located on the cell membrane and that it reduced intracellular Ca^2+^ levels and reactivated P53 and PTEN. We further demonstrated that NCX2 impaired cell invasion through the HIF-1α pathway in glioma. The results indicated that NCX2 plays a key role in glioma formation and tumor invasion functionality.

## Introduction

Glioma is the most common and malignant primary tumor of the central nervous system (Reifenberger et al. 2017). Due to high degrees of proliferation, invasion, and angiogenesis, glioma is extremely refractory to surgical resection, chemotherapy, and radiotherapy. The prognosis in patients with tumors is associated with many biological and clinical characteristics, such as stage of the disease, patient’s age, and genetic and epigenetic molecular features of the tumor. Solid glioma is hypoxic, and hypoxia and its regulator hypoxia-inducible factor 1 (HIF-1) play a key role in tumor invasion and angiogenesis (Domènech et al. 2021). In addition to oxygen, the activation of oncogenes, such as EGFR, P53, and PTEN, can affect HIF-1 expression (Domènech et al. 2021; Lv et al. 2017; Ravi et al. 2000). HIF-1 consists of two subunits, HIF-1α and HIF-1β. The expression and activation of HIF-1α determine the functionality of HIF-1. The regulation occurs at the levels of mRNA expression, protein expression, and nuclear localization. In normoxic conditions, HIF-1α can be hydroxylated by prolyl hydroxylases (PHDs), leading to ubiquitylation and proteasomal degradation. However, PHDs are no longer active in hypoxia; HIF-1α and HIF-1β bind together and translocate into the nucleus to promote cell proliferation, energy metabolism, invasion, and angiogenesis (Palazon et al. 2014; Semenza 2012).

**Fig. 1:**
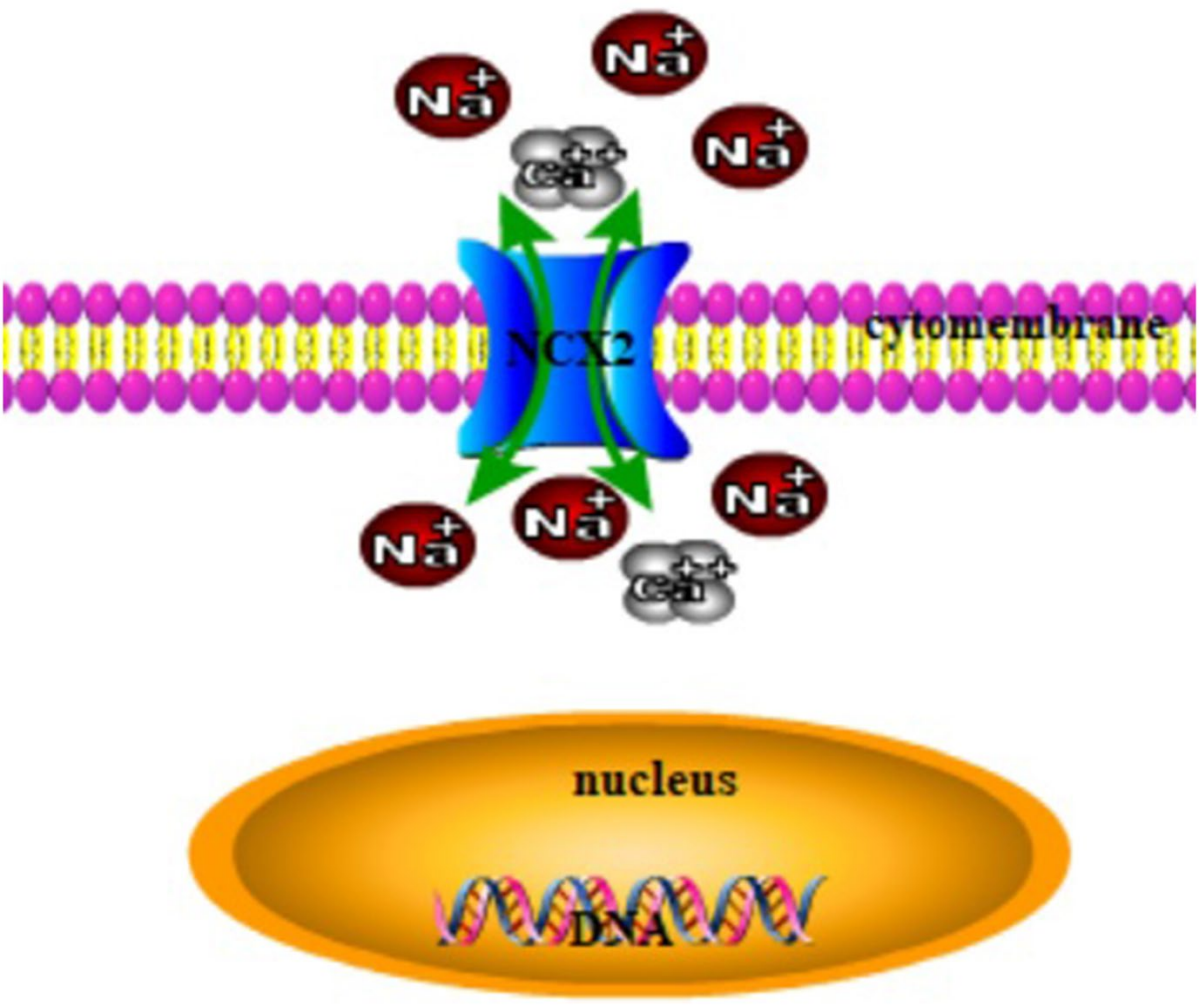
NCX2 cell sublocalization dimensional views. Na^+^ and Ca^2+^ are transported bidirectionally by NCX2

Loss of heterozygosity on the arm of 19q13 is commonly found in human malignant glioma, especially in oligodendroglioma (Mizoguchi et al. 2011). This suggests that at least one tumor suppressor gene important for the development of gliomas is located in 19q13. SLC8A2/NCX2 encodes Na^+^/Ca^2+^ exchanger 2 (NCX2), which is a transmembrane protein that mediate Ca^2+^ efflux/influx to regulate the cytosolic Ca^2+^ homeostasis in many cell types (Fig. 1), and is located in 19q13 (Annunziato et al. 2004; Qu et al. 2010). NCX2 plays a neuroprotective role in the ischemic adult brain and is present in the brain and skeletal muscle. It has been reported that NCX2 is silenced in the glioma samples and the glioma cell lines U87, U343, and U343Cl 2:6, as a result of DNA methylation (Qu et al. 2010). Ca^2+^-mediated signaling pathways, such as protein kinase C (PKC), play a key role in tumorigenesis and tumor progression (Kawano et al. 2021). Our previous study showed that NCX2 inhibited the tumorigenicity of U87 cells in nude mice (Qu et al. 2017). These observations suggested that NCX2 might be a tumor suppressor, which is silenced in gliomas. Malignant tumors often experience a hypoxic microenvironment. To more accurately understand the effect of NCX2 on glioma, we constructed a hypoxic microenvironment and used lentivirus plasmid to turn on the expression of NCX2 in glioma cell line U87, to further understand the role and molecular mechanism of NCX2 in glioma cells in vitro.

## Materials and Methods

### Cell Culture and Hypoxia

The human glioma cell lines U87MG were purchased from the Cell bank of the Chinese Academy of Science. All cells were cultured in Dulbecco’s modified Eagle’s medium (DMEM/HG, Gibco, Invitrogen, Life Technologies, Spain) supplemented with 10% fetal bovine serum (FBS, Gibco, Life technologies, USA) and 1% penicillin and streptomycin in a standard humidified incubator at 37 °C with 5% CO_2_.

For the exposure to hypoxia, the cells were placed in a Modular Incubator Chamber (Thermo, USA), flushed with 2% O_2_, 5% CO_2_, and 93% N_2_, and incubated at 37 °C.

### Lentivirus Production and Infection of the Glioma Cells

Recombinant lentivirus plasmid expressing the gene NCX2 (GenePharma Co., Ltd, Shanghai, China) was used to generate Lenti-NCX2-IRES-EGFP. Transfection was conducted as previously described. In brief, the cells at 50–60% confluence were transfected using a lentivirus reagent. Transfection complexes were prepared in line with the manufacturer’s instructions. U87-NCX2 cells were U87 cells stably transfected with NCX2, and the control U87-NC cells were U87 cells stably transfected with Lenti-EGFP. Transfection was able to upregulate NCX2 expression in U87 cells based on reverse-transcription polymerase chain reaction (RT-PCR) and western blot analysis.

### Establishment of U87 with Low Expression of HIF-1α

For HIF-1a knockdown and a negative control, small interfering RNAs (siRNAs) targeting HIF-1α and transfection of green fluorescent protein (GFP) were used. Logarithmically grown U87 cells were taken, and when the cell confluence reached approximately about 70%, the cells were transiently transfected with Lipofectamine™ 3000 according to manufacturer’s instructions. After transfection, they were divided into the interference group (HIF-1α-siRNA), control interference group (control-siRNA), and untreated group.

### Immunofluorescence

The cells were plated in 6-well plates, cultured in DMEM including 10% FBS, washed thrice with ice-cold phosphate-buffered saline (PBS, Boster, Wuhan, China), and fixed in 4% paraformaldehyde for 20 min. PBS containing 0.3% Triton X-100 (PBS-T) was added to each well. The cells were blocked with 1% bovine serum albumin (BSA, Sunshine biotechnology, NanJing, China) in PBS-T for one hour at room temperature, and incubated with anti-SLC8A2/NCX2 monoclonal antibody (1:500 dilution, MBL, Japan) at 4 °C overnight. We added Cy3-labeled goat anti-rabbit IgG in an immunofluorescence staining kit (Beyotime, China). Before mounting, the cells were stained with 1’,6-diamino-2-phenylindole (DAPI, Beyotime, China). Then, they were viewed and photographed under a fluorescence microscope (Zeiss Observer A1, Germany).

### Ca^2+^ Imaging

The cells were cultured in a hypoxic microenvironment. They were plated in confocal dishes at 20 °C for 60 min in hypoxia with 5 μM X-Rhod-1 AM (Life Technologies, USA) provided as a cell-permanent acetoxymethyl ester, and imaged in Hank’s balanced salt solution (HBSS, Gibco, USA) with 0.2% Pluronic F-127 (Life Technologies, USA). In line with the manufacturer’s instructions, before fluorescence measurements were commenced, the cells had been washed to remove any dye nonspecifically associated with the cell surface and then incubated for a further 30 min. X-Rhod-1 was excited at 568 nm. Ca^2+^ signals in U87-NCX2 cells were imaged under confocal laser scanning microscopy (Leica, Germany) with the same parameters as control cells.

### Cell Proliferation

The number of viable cells was assayed using Cell counting kit-8 (CCK-8, Dojindo MD) as per the manufacturer’s protocol. The cells were seeded into the 96-well plates (3000 cell/well). The cells were placed in hypoxia at 37 °C. After 24 h, 10 µL CCK-8 solution was added to each well. After 2 hours of incubation in hypoxic conditions, the optical density at 450 nm was measured using an enzyme-linked immunosorbent assay reader. GraphPad Prism 5 was used to analyze the results.

### Migration and Invasion Assay

Cell migration and invasion were evaluated in 24-well transwell inserts with 8 µm-large pores (Corning, NY, USA). The cells were suspended in serum-free medium, and added to the upper chambers at a density of 2 × 10^4^ cells per well. DMEM with 10% FBS was added to the lower chambers. After 36 h of incubation in hypoxia at 37 °C, we removed the non-migrated cells on the upper surface, and the cells on the lower side of the filter surface were fixed with crystal violet cell colony staining kit (GENMED, Boston, USA). The stained cells were counted in 5–8 fields per filter at 200 × magnification under an EVOS microscope. The cell invasion assessment was the same as previously described, except that the upper chambers of the transwell inserts were covered with Matrigel (40 µL/well, BD Bioscience, Bedford, MA).

### RT-PCR

Total RNA in hypoxic conditions was extracted from the cultured cells by TRIzol reagent (Life Technologies, USA). The isolated RNA was reverse-transcribed into complementary DNA using RevertAidTM First Strand cDNA Synthesis Kit (Fermentas, Shanghai, China).

Gene primers used were as follows: NCX2: 5′-CTGACGGTGTTCTGGAAGGT-3′ (forward), 5′-CTGAGATCCTACCCCACCAA-3′ (reverse); HIF-1α:5′-CCTGAGCCTAATAGTCCC-3′ (forward), 5′-GGTGGCATTAGCAGTAGG-3′ (reverse); MMP2: 5′-CAGGCTCTTCTCCTTTCACAAC-3′ (forward), 5′-AAGCCACGGCTTGGTTTTCCTC-3′ (reverse); MMP7: 5′-GTCTCTGGACGGCAGCTATG-3′ (forward), 5′-GATAGTCCTGAGCCTGTTCCC-3′(reverse); MMP14: 5′-TCCAGCAACTTTATGGGGGT-3′ (forward), 5′-TTCCCGTCACAGATGTTGGG-3′ (reverse); TIMP1: 5′- ACCAGACCACCTTATACCA-3′ (forward), 5′-CATTCCTCACAGCCAACA-3′ (reverse); VEGF: 5′-CACATAGGAGAGATGAGCTTC-3′ (forward), 5′-CCGCCTCGGCTTGTCACAT-3′ (reverse); PAI-1: 5′-AAGGACTGTTCCTGTGGGGT-3′ (forward), 5′-AGCCACTGGAAAGGCAACAT-3′ (reverse); GAPDH: 5′-GGAAGGTGAAGGTCGGAGTC-3′ (forward), and 5′-GAGGCATTGCTGATGATCTTGA-3′ (reverse). The PCR conditions were as follows: initial denaturation at 94 °C for 5 min was followed with 25–30 cycles of 94 °C for 30 s, 52–55 °C for 30 s, and 72 °C for 30 s, and a final extension at 72 °C for 7 min. RT-PCR products were separated on 1.5% agarose gels stained with GelRed (Biotium, CA, USA), and were visualized under ultraviolet light. The results were analyzed by Quantity One software (Bio-Rad). GAPDH was used as an internal reference.

### Western Blotting

For total protein extraction, the cells in hypoxic conditions were washed with precooled PBS solution, and whole-cell proteins were extracted by incubation with the radio immuno precipitation assay (RIPA) buffer (Beyotime, China). For separation of nuclear and cytoplasmic proteins, NE-PER Nuclear and Cytoplasmic Extraction Reagents (Thermo Scientific, Rockford, USA) were used. Protein concentrations were determined by BCA Protein Assay Kit (Beyotime, China). A total of 50 µg of protein was electrophoresed on an 8% or 10% SDS-PAGE and transferred to nitrocellulose membranes. The membranes were blocked with 5% nonfat dry milk in TBST including 0.01% Tween 20 (Beyotime, China) for one hour at room temperature, and then incubated with 1:1000 dilution of anti-SLCA82/NCX2 monoclonal antibody (MBL, Japan) at 4 °C overnight. Anti-HIF-1α (rabbit polyclonal, 1:1000) and PTEN (rabbit polyclonal, 1:1000) were purchased from Abcam company. Anti-P53 (rabbit polyclonal, 1:000), anti-PKCα (rabbit polyclonal, 1:800), and anti-PKCβ (rabbit polyclonal, 1:900) were purchased from Sangon (Shanghai, China). We added secondary antibody horseradish-peroxidase (HRP)-conjugated anti-mouse IgG (1:9000, MultiSciences Biotech, Hangzhou, China) and incubated for an hour at 37 °C. Specific bands were visualized with Enhanced Chemiluminescence Detection Kit for HRP (Biological Industries, kibbutz Beit Haemerk, Israel) using Kodak X-OMAT LS film (Eastman Kodak, Rochester, NY). Quantitative data were obtained using Quantity One software.

### Statistical Analysis

Statistical analyses were made using Statistical Package for the Social Sciences (SPSS) software, version 19.0 (Chicago, IL, USA). Data were represented as the mean values with standard deviations (SD). All data were analyzed by Student’s *t* test or one-way ANOVA. Differences were considered statistically significant at *P* < 0.05.

## Results

### NCX2 and HIF-1α Expression in U87

**Fig. 2:**
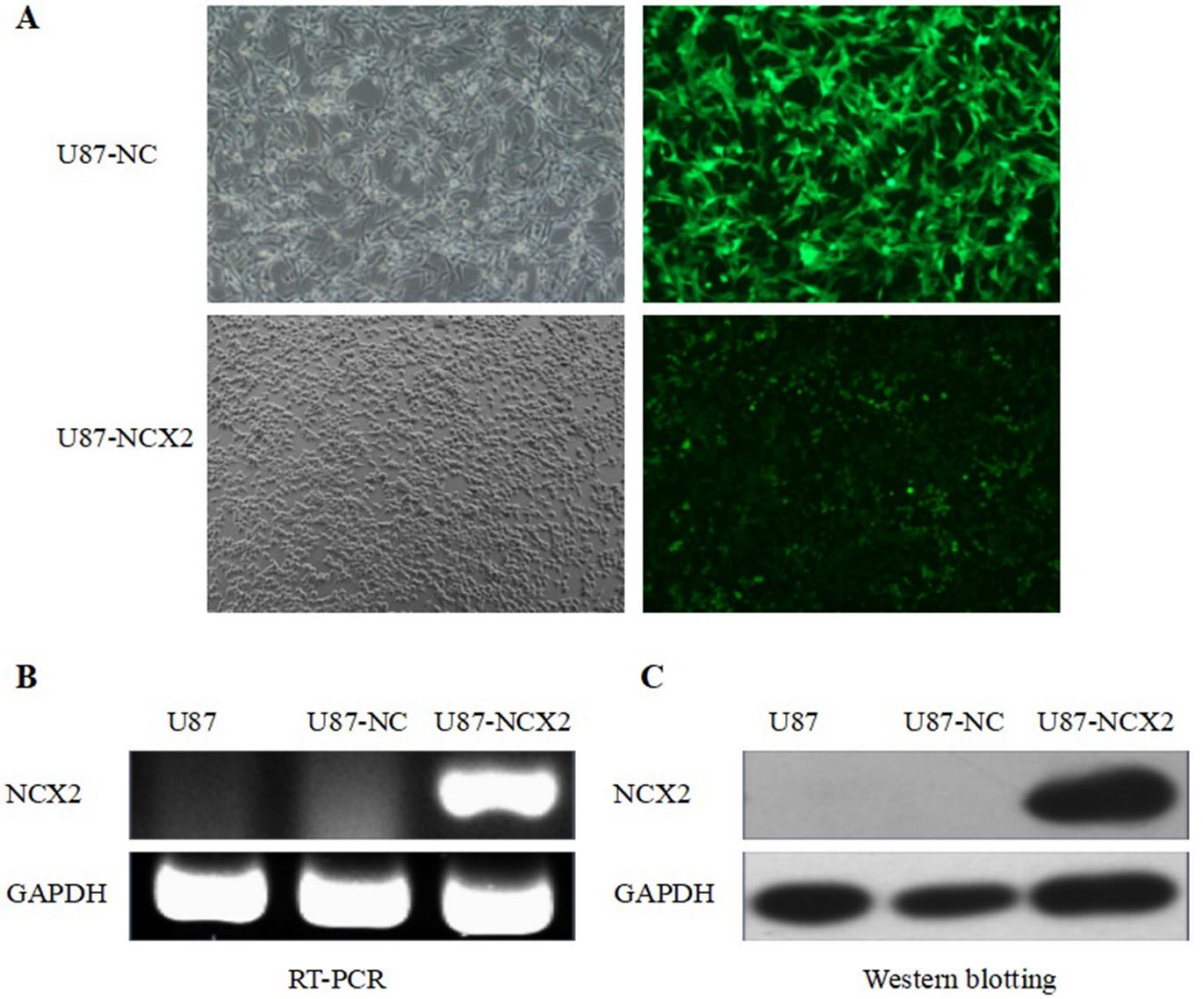
Lentiviral vector transduction and NCX2 expression. **A** Lentiviral vector-transduced cells. U87-NC and U87-NCX2 were imaged under fluorescence microscope at × 400. **B** RT-PCR for NCX2 mRNA expression in U87MG. GAPDH was used as an internal reference. **C** Western blot analysis for NCX2. After transfection with NCX2, proteins from U87-NC and U87-NCX2 were collected and western blotting showed an increase in NCX2 protein. GAPDH was used as a loading control. *P < 0.01

**Fig. 3:**
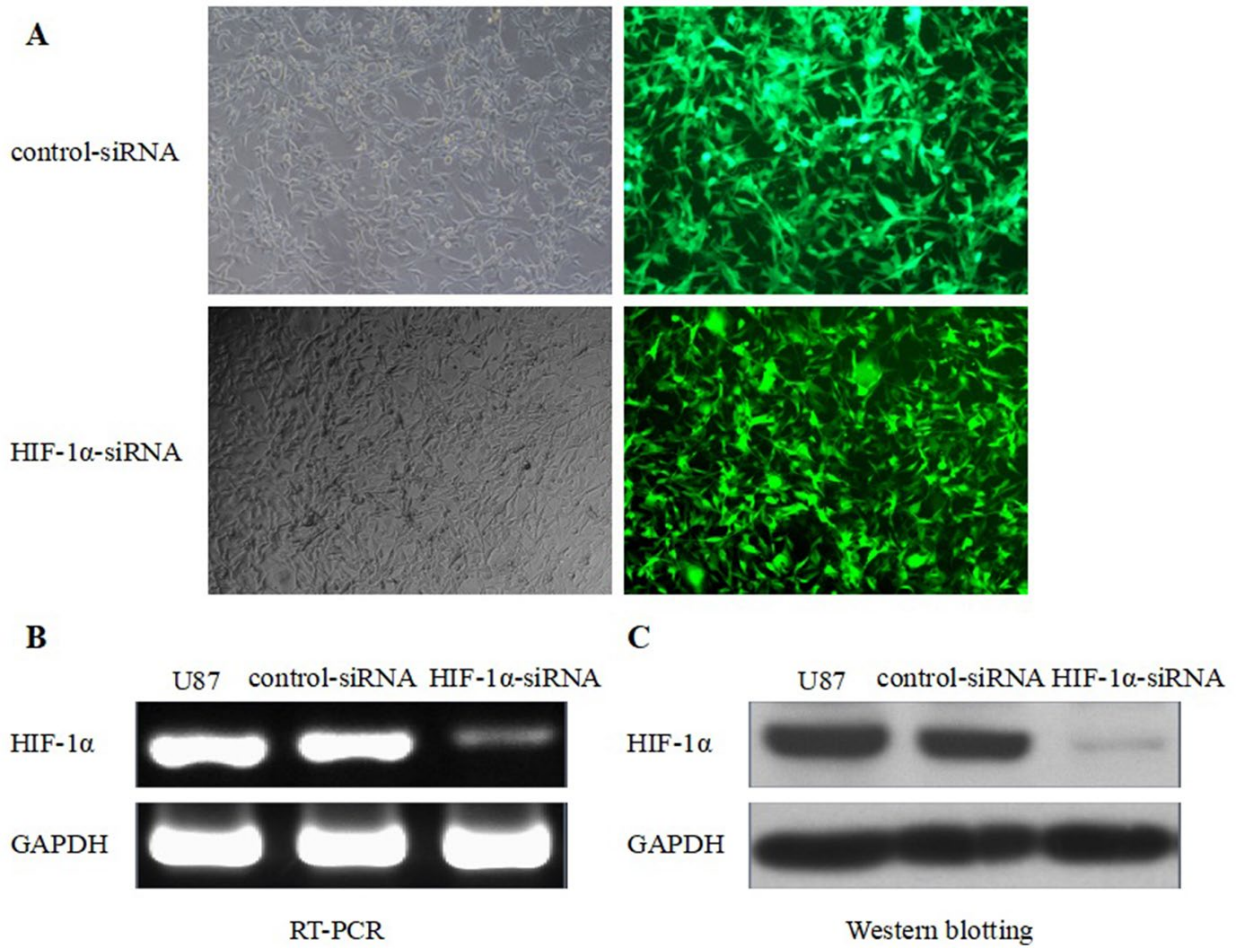
HIF-1α knockdown and HIF-1α expression. **A** small interfering RNAs (siRNAs) cells. Control-siRNA and HIF-1α-siRNA were imaged under fluorescence microscope at × 400. **B** RT-PCR for HIF-1α mRNA expression in U87MG. GAPDH was used as an internal reference. **C** Western blot analysis for HIF-1α. After transfection with siRNAs, proteins from Control-siRNA and HIF-1α-siRNA were collected and western blotting showed a decrease in HIF-1α protein. GAPDH was used as a loading control. *P < 0.01

RT-PCR was used to analyze the expression of NCX2 and HIF-1α in glioma cell U87MG. As shown in (Figs. 2B, 3B) NCX2 was not expressed in U87, but HIF-1α was expressed in U87, normalized to *β*-tubulin. To understand the role of NCX2 in glioma cell physiology under hypoxic conditions, we constructed the U87 cells stably transfected with NCX2 (Fig. 2A). The level of expression of NCX2 was significantly upregulated in U87-NCX2 compared with U87MG and U87-NC (negative control), verifying that U87 cells stably transfected with NCX2 were constructed (Fig. 2B, C). To understand the effect of HIF-1α in glioma, we constructed glioma cell U87MG with low expression of HIF-1α (Fig. 3A). The expression of HIF-1α mRNA and total HIF-1α protein was significantly decreased in the HIF-1α-siRNA group, compared with the untreated group and control-siRNA group (Fig. 3B, C). HIF-1α in HIF-1α-siRNA nucleus was barely detectable in hypoxia (data not listed).

### ***Ca***^***2***+^***Imaging and Immunofluorescence***

**Fig. 4:**
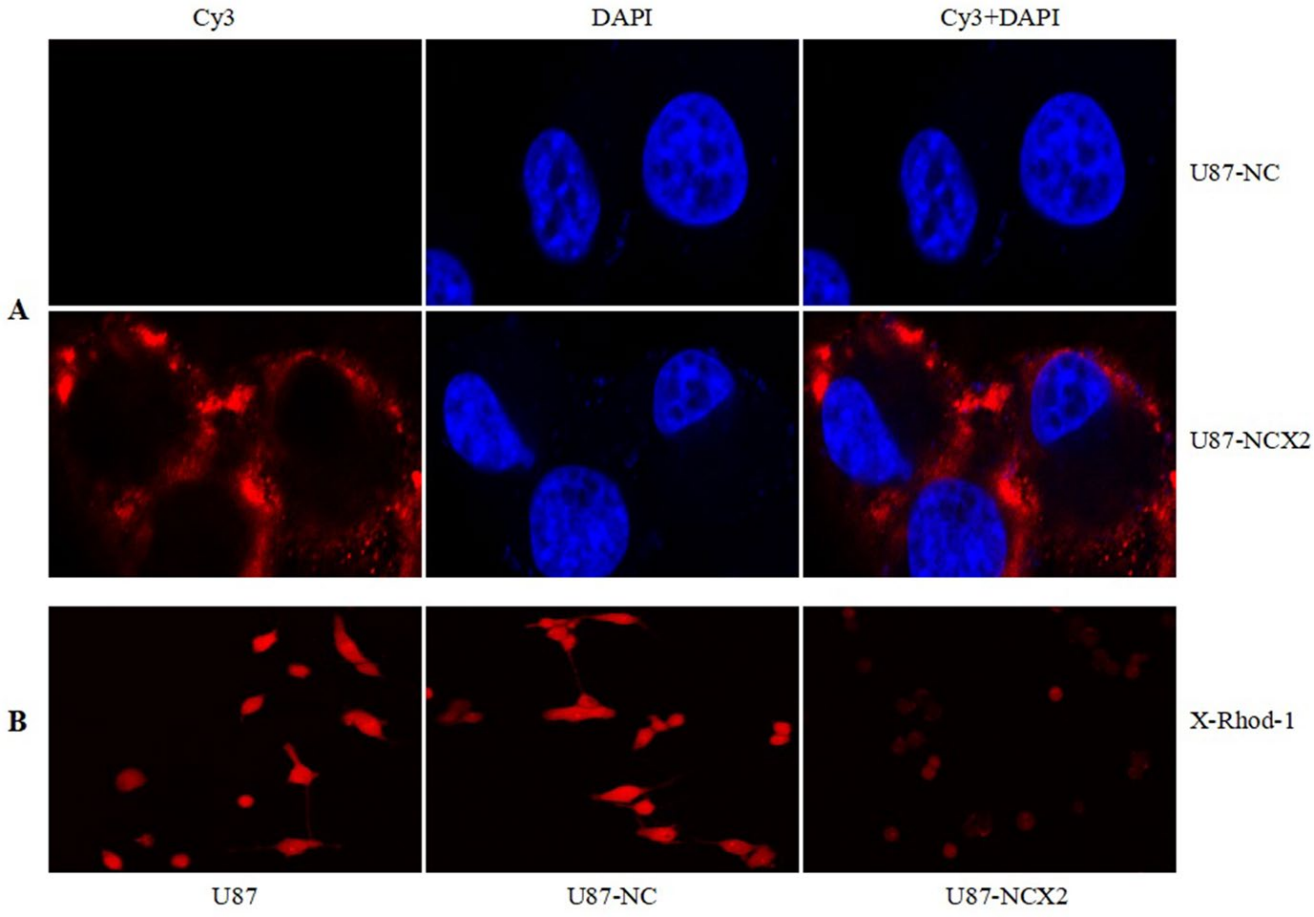
NCX2 detected on cell membrane and regulated Ca^2+^. To detect the localization of NCX2 in transfected U87 cells, immunofluorescence was used. **A** U87-NCX2 showed the upregulation of NCX2 (red fluorescence). The cells were counterstained with DAPI (blue fluorescence) to make the nucleus visible. **B** NCX2 reduced intracellular calcium ions

To determine the subcellular localization of NCX2 in U87 cells transfected with the NCX2 gene, we conducted an immunofluorescence study. We showed that NCX2 was detected on the cell membrane (Fig. 4A). To evaluate the role of NCX2 in controlling the intracellular Ca^2+^, X-Rhod-1, and confocal laser scanning microscopy were used to examine the changes of Ca^2+^ levels. As shown in Fig. 4B, the Ca^2+^ level in U87-NCX2 cells was significantly reduced.

### NCX2 Promotes U87 Cells’ Proliferation in Hypoxia as Shown by CCK8

**Fig. 5:**
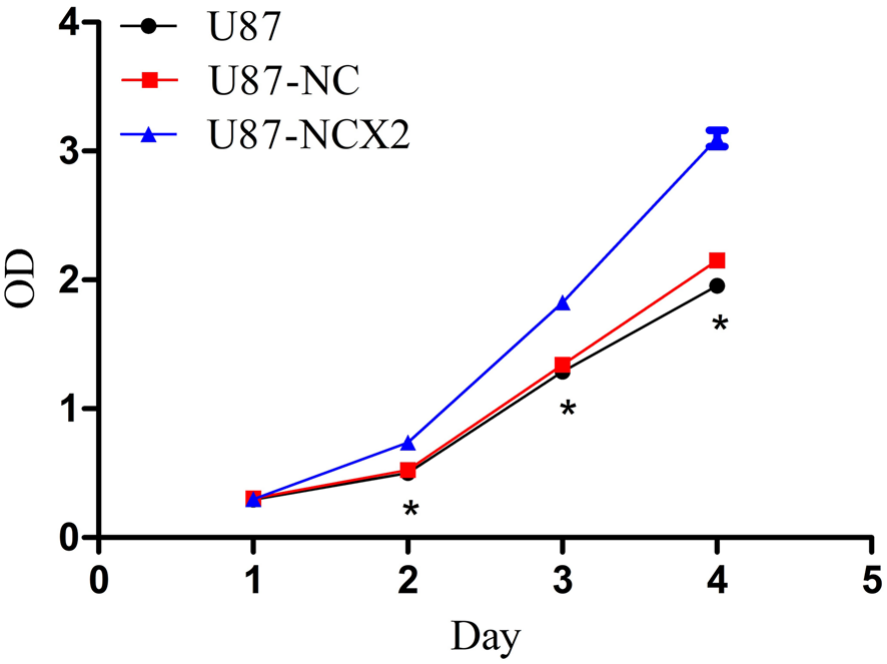
Growth curve of U87-NC and U87-NCX2 under hypoxic conditions. Hypoxia insignificantly inhibited U87-NCX2 cell proliferation in the early 48 h and slightly promoted cell proliferation after 48 h. (*P < 0.01, *n* = 5)

It has been reported that hypoxia can inhibit U87 cell proliferation (Li et al. 2013). The correlation between NCX2 and glioma cell proliferation in an anoxic micro environment has not been reported. To evaluate whether NCX2 is involved in the control of proliferation of U87 cells in hypoxia, a CCK8 assay was used. After the culture for 4 days in hypoxia, over expression of NCX2 in U87 cells promoted their proliferation compared with negative control U87-NC (Fig. 5). These results suggested that NCX2 promoted the proliferation of U87 cells in vitro in hypoxic conditions; hypoxia insignificantly inhibited U87-NCX2 cell proliferation in the early 48 h and slightly promoted cell proliferation after 48 h.

### Role of NCX2 and HIF-1α in Invasion and Migration of U87 Cells in Hypoxia

**Fig. 6:**
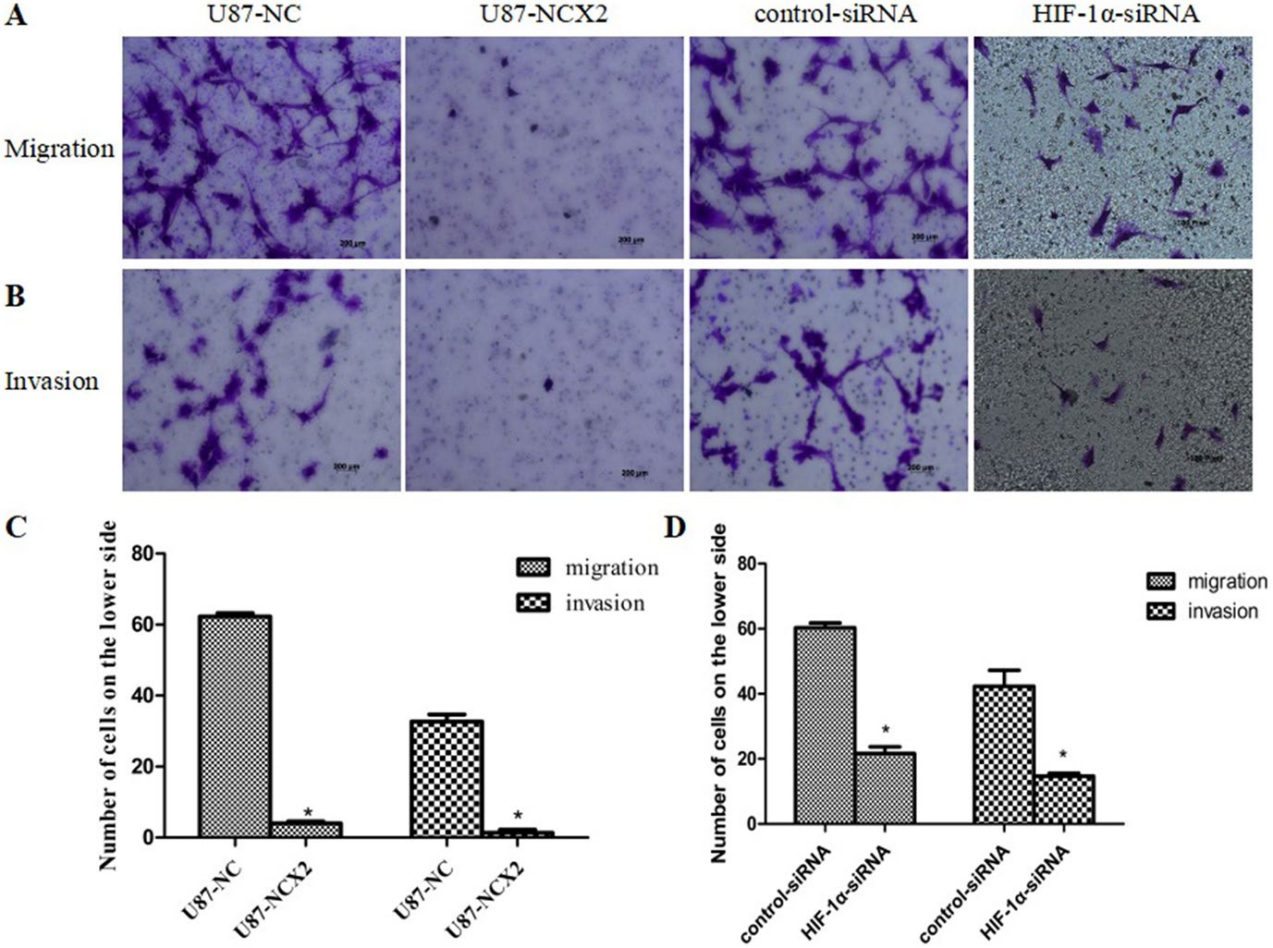
NCX2 and HIF-1α inhibited invasion and migration in vitro under hypoxic conditions. **A** Cells migration after hypoxia for 36 h. **B** Cells invasion after hypoxia for 36 h. **C, D** Comparison of cells migration (left) and invasion (right) under hypoxia for 36 h (migration) and 36 h (invasion) (*P < 0.01)

Cells invasion and migration are important features of glioma, and hypoxia can increase the invasion and migration ability of U87 cells (Li et al. 2013). To study the role of NCX2 in the invasion or migration of U87 cells under hypoxic conditions, we analyzed the ability of stable transfectants expressing NCX2 to migrate through matrigel-coated or non-matrigel-coated in hypoxia. In hypoxic conditions, NCX2 significantly reduced the invasion and migration of the cells compared with the control group (Fig. 6A–C). These results suggest that NCX2 suppresses glioma cells’ invasion and migration in a hypoxic environment.

Meanwhile, glioma cell U87MG with a low expression of HIF-1α was constructed, and its invasion and migration ability in a hypoxic microenvironment was detected. Compared with the control-siRNA group, the invasion and migration ability of the HIF-1α-siRNA group was significantly inhibited in the hypoxic microenvironment (Fig. 6A, B, D).

### NCX2 Inhibits PKC and Activates P53 and PTEN Expression

**Fig. 7:**
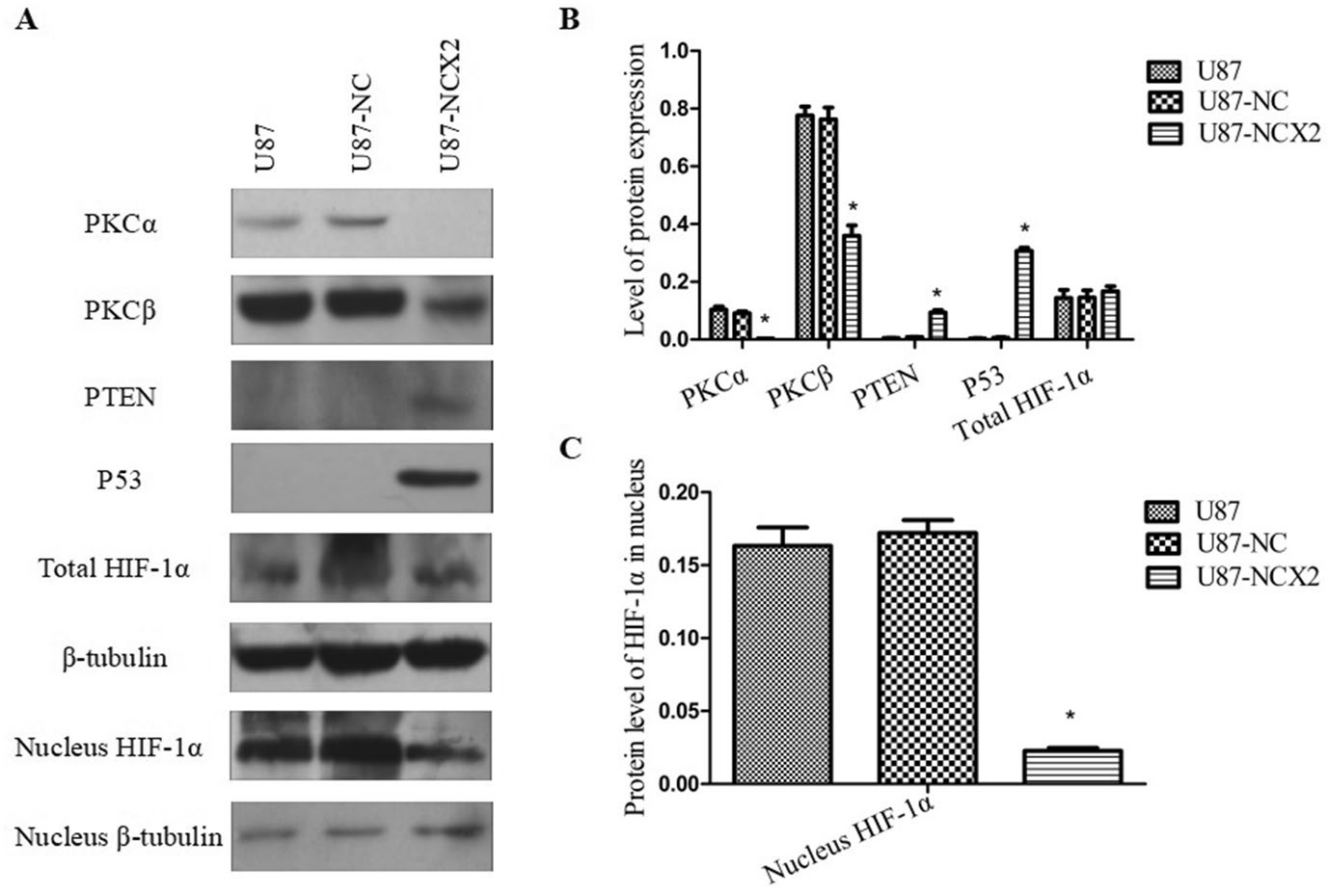
The effect of NCX2 on the protein expression levels of PKCα, PKCβ, PTEN, and P53. To determine the effect of NCX2 on the HIF-1α pathway, total HIF-1α and nucleus HIF-1α protein were extracted. There was a significant difference between U87-NCX2 and U87-NC. *β*-tubulin was used as control (*P < 0.01)

Ca^2+^ contributes to motility, angiogenesis, and cell cycle (Liang et al. 2021). Previous studies have reported that NCX2 contributes to the Ca^2+^ influx required for the activation and targeting of PKCα, and Ca^2+^ could mediate the transactivation of P53 (Andrikopoulos et al. 2011a; Gogna et al. 2012). The expression levels of PKCα, PKCβ, P53, and PTEN were examined under hypoxic conditions in U87, U87-NC, and U87-NCX2. The data showed that NCX2 reduced PKCα and PKCβ in hypoxia, but activated P53 and PTEN (Fig. 7A, B).

### NCX2 Reduces the Activation of the HIF-1 Pathway in U87 Cells

Hypoxia can directly regulate gene transcription by binding HIF-1α. High levels of HIF-1α are expressed in many cancers, and they correlate with tumor growth and migration. To examine whether NCX2 overexpression affects HIF-1α expression in glioma, HIF-1α protein in cells under hypoxia was analyzed, where *β*-tubulin was used as an internal control. Total HIF-1α and HIF-1α in the nucleus were determined by western blot analysis. As shown in Fig. 7, total HIF-1α was not altered in the three groups (Fig. 7A, B). However, HIF-1α protein in the nucleus was reduced (Fig. 7A, C). These data showed that NCX2 inhibited HIF-1α translocation into the nucleus.

### NCX2 Reduces the Downstream Genes Regulated by HIF-1α

**Fig. 8:**
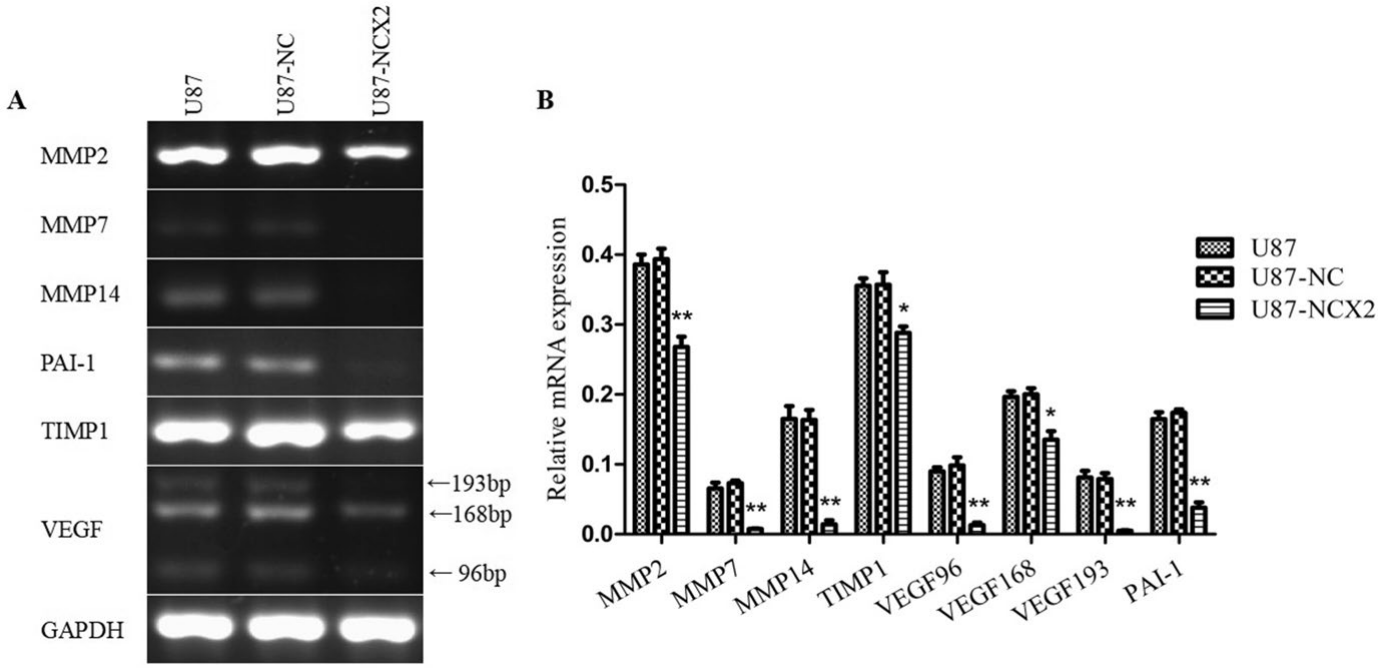
VEGF96, product length = 96 bp; VEGF168, product length = 168 bp; VEGF193, product length = 193 bp. U87-NC and U87-NCX2 cells were subjected to hypoxia. The mRNA expression levels of MMP2, MMP7, MMP14, TIMP1, VEGF, and PAI-1 were analyzed by RT-PCR. GAPDH was used as an internal control (*P < 0.05, **P < 0.01)

Invasion depends on complex pathways under hypoxic conditions. Hypoxia can directly regulate gene transcription by binding HIF-1. MMPs, TIMP1, VEGF, and PAI as HIF-1 downstream genes are regulated by HIF-1. NCX2 could inhibit HIF-1α translocation into the nucleus, suggesting that NCX2 may be the upstream gene that regulates HIF-1α. To determine the relationship between NCX2 and glioma aggressiveness and angiogenesis in hypoxia, RT-PCR was used to analyze the mRNA levels of MMP2, MMP3, MMP7, MMP14, TIMP1, VEGF, and PAI-1 to detect whether HIF-1 led to those gene changes. GAPDH was used as an internal control. As shown in Fig. 8, MMP2, MMP7, MMP14, VEGF, TIMP1, and PAI-1 levels were reduced in hypoxia in U87-NCX2.

## Discussion

The loss of 19q13 is a common genetic alteration in gliomas that is associated with clinical behavior and survival. Moreover, it has been reported that 19q13 loss of heterozygosity is associated with poor survival in neuroblastoma (Mora et al. 2001; Cairncross et al. 2006). All these findings suggest the location of the tumor suppressor gene (or genes) in 19q13.

SLC8A2/NCX2 belongs to the Na^+^/Ca^2+^ exchanger gene family SLC8, which mediates Ca^2+^ transport to regulate the cytosolic Ca^2+^. It is located on 19q13.32. Ca^2+^ homeostasis controls cellular motility, angiogenesis, transcription, cell cycle, apoptosis, and other processes. Distinct NCX isoform/splice variants regulate blood pressure, immune response, neurotransmitter secretion, and insulin secretion (Annunziato et al. 2004). NCX2 is expressed in the brain and silenced in glioma because of DNA methylation. RT-PCR was used to analyze the significantly reduced expression of NCX2 and HIF-1α in glioma cell lines (Figs. 2B, 3B). Despite the recent progress in understanding the role of NCX2 in health, its molecular and cellular mechanisms in tumors are not yet clear. Ca^2+^ influx through NCX is required for the activation and targeting of PKCα to the plasma membrane, an essential step for VEGF-induced ERK1/2 phosphorylation and angiogenesis (Andrikopoulos et al. 2011b). This is consistent with our previous finding that NCX2 can inhibit glioma proliferation and growth through the ERK signaling pathway. Patients with P53 deletion exhibit higher Ca^2+^ in serum (Chang et al. 2005). P53 silencing can inhibit Ca^2+^ release; the intracellular Ca^2+^ is released by stabilizing the P53–p300 complex and recruiting P53 to P53 promoter in wild-type p53 cells. PKC contains a regulatory domain located in the N-terminal region: diacylglycerol (DAG, C1 domain) and Ca^2+^ (C2 domain). Conventional PKC (α, βI, βII, and γ) is activated by both Ca^2+^ and diacylglycerol (DAG); however, novel PKC isozymes (δ, ε, θ, and η) only require DAG for their activation (Kawano et al. 2021). In this study, X-Rhod-1 was used for detecting Ca^2+^. We found that the cellular Ca^2+^ level was significantly reduced compared with the control group (Fig. 4B). The expression of PKC(α,β) activated by Ca^2+^ was analyzed; we showed that PKCα and PKCβ were significantly reduced (Fig. 7). U87 cells are wild-type P53 cells. We found that NCX2 was able to activate P53 re-expression (Fig. 7). PTEN regulates intracellular Ca^2+^, and Ca^2+^ may regulate subcellular localization of PTEN (Bononi and Pinton 2015; Minaguchi et al. 2006), suggesting that PTEN has a close relationship with Ca^2+^. We also examined whether changes in Ca^2+^ can induce PTEN expression. The results revealed that changes in Ca^2+^ were able to activate PTEN expression.

Hypoxia is an important aspect of the glioma microenvironment given that it induces angiogenesis, tumor growth and invasion, and resistance to radiotherapy and chemotherapy (Sullivan and Graham 2007). HIF-1 plays a key role in hypoxia-related physiology and pathology. HIF-1α is commonly expressed in tumors, but the role of HIF-1α in cancer is not fully understood. The role of hypoxia resulting in HIF-1α in glioma migration is well recognized. During hypoxia, HIF-1α and HIF-1β bind together and make a complex with p300/CREB binding protein (CBP) to translocate into the nucleus. Our data showed that the overexpression of NCX2 reduced the migration of human glioma cells in vitro in hypoxic conditions (Fig. 6). HIF-1α inhibited the invasion and migration ability of U87 in the hypoxic microenvironment (Fig. 6). P53 can affect HIF-1α expression. We examined the total HIF-1α and HIF-1α in the nucleus. The results showed that the overexpression of NCX2 did not affect the total HIF-1α protein expression; however, it was associated with the reduction of HIF-1α in the nucleus (Fig. 7). These findings suggest that P53 may compete with HIF to bind p300, thereby inhibiting HIF-1α translocation into the nucleus, rather than directly promoting HIF-1α degradation.

Some studies have shown that HIF-1α translocates to the nucleus to regulate angiogenesis and promote tumor growth and invasion. MMP2, MMP7, MMP14, and VEGF are upregulated by HIF-1-dependent pathways in hypoxia (Domènech et al. 2021; Meter et al. 2004). PAI-1 is an inhibitor of tissue (t-PA) and urokinase (u-PA) plasminogen activators, and it is increased in hypoxia by an HIF-1-mediated pathway. Unlike in physiological conditions, PAI-1 in glioma regulates invasion and release of VEGF leads to increased angiogenesis (Hjortland et al. 2004). TIMP1, an inhibitor of MMPs, may contribute to glioma malignancy when upregulated (Groft et al. 2001). MMP2, MMP7, MMP14, TIMP1, and PAI-1—HIF-1 downstream genes—were analyzed by RT-PCR. Those genes in U87-NCX2 were decreased in hypoxia, compared with the control group (Fig. 8). Based on the results, we plan to conduct related studies on glioma angiogenesis induced by NCX2 under hypoxic conditions.

## Conclusions

**Fig. 9:**
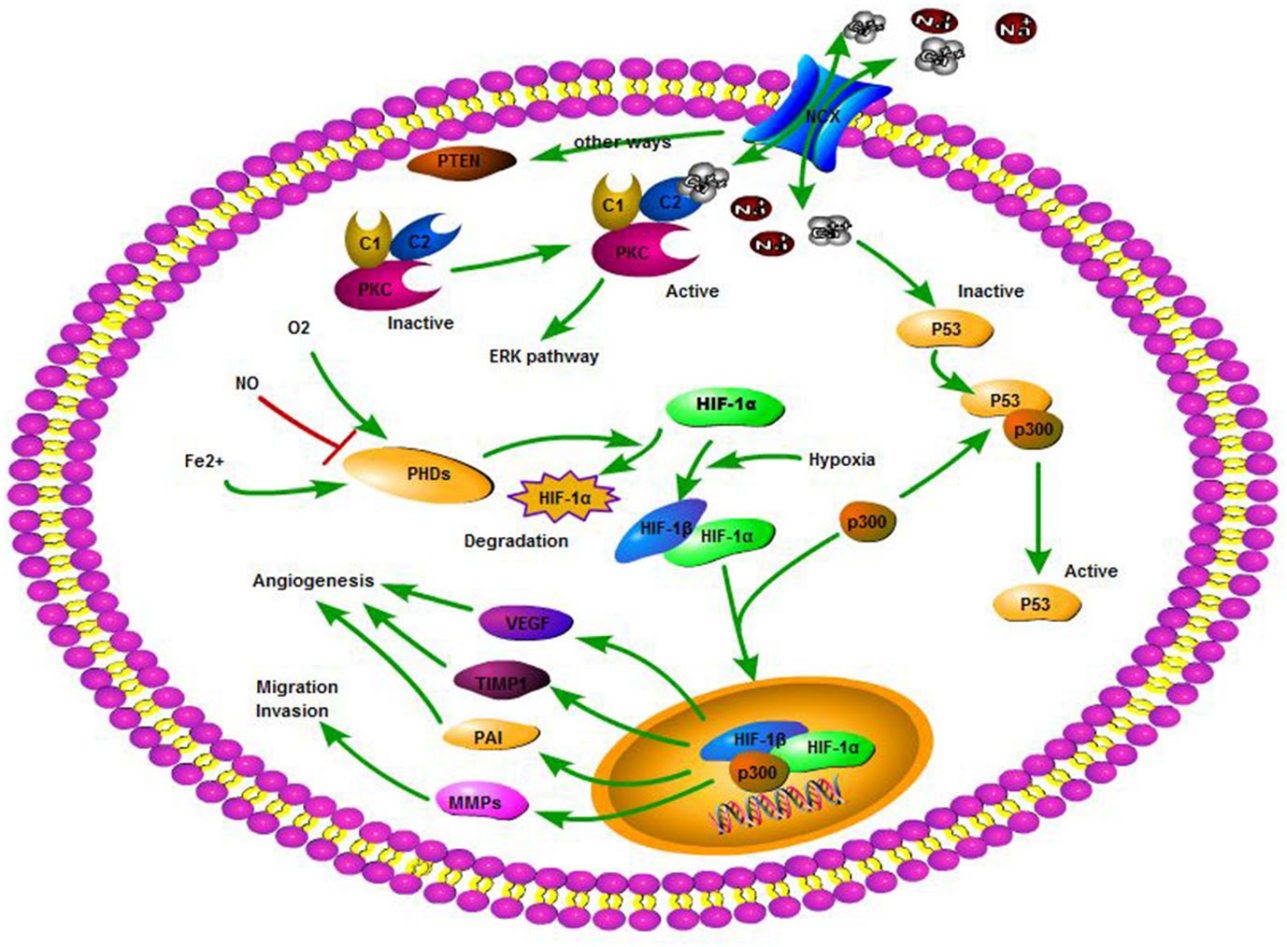
From KEGG and Biocarta database and our study, we established a cell pathway model of NCX2 regulation in U87 cells. Ca^2+^ efflux/influx through NCX2 to increase/decrease the Ca^2+^ in the cell. PKCs or wild-type P53 are activated by increased intracellular Ca^2+^. PKC expression to achieve a regulation of the ERK pathway. When P53 is activated, it competes with HIF-1α to bind p300 and inhibit HIF-1α nuclear translocation affecting cell proliferation, energy metabolism, invasion, and angiogenesis. Ca^2+^ can also regulate the expression of PTEN by other ways to affect the HIF-1 pathway

We presented our research in a model diagram (Fig. 9). We found that NCX2 in glioma could reduce intracellular Ca^2+^, which led to inactive PKCs and active PTEN and P53. Those genes could reduce glioma cells invasion and migration by the HIF-1α pathway. Furthermore, malignant tumor proliferated rapidly and required more energy, thereby resulting in the tumor’s hypoxic microenvironment. Although NCX2 promoted the proliferation of glioma cells in hypoxic conditions, it could not obtain sufficient energy to induce apoptosis due to the inhibition of cell invasion by NCX2. This explained why NCX2 promoted tumor proliferation in hypoxia, but could not induce tumors in nude mice.
